# A novel *LAMP2* p.G93R mutation associated with mild Danon disease presenting with familial hypertrophic cardiomyopathy

**DOI:** 10.1002/mgg3.941

**Published:** 2019-08-28

**Authors:** Jing Xu, Lu Wang, Xiangdong Liu, Qiming Dai

**Affiliations:** ^1^ Department of Clinical Laboratory, ZhongDa Hospital Southeast University Nanjing P. R. China; ^2^ Institute of Life Science Southeast University Nanjing P. R. China; ^3^ Department of Cardiology, ZhongDa Hospital Southeast University Nanjing P. R. China

**Keywords:** Danon disease, exome sequencing, hypertrophic cardiomyopathy, *LAMP2*, mutation

## Abstract

**Background:**

Danon disease (DD) is an X‐linked dominant multisystem disorder that is associated with cardiomyopathy, skeletal myopathy, and varying degrees of intellectual disability. It results from mutations in the lysosome‐associated membrane protein 2 (*LAMP2*) gene.

**Methods:**

Herein, a proband with a mild DD case presenting with a familial hypertrophic cardiomyopathy (HCM) phenotype and additional family members were evaluated. Exome sequencing and Sanger sequencing were performed to explore the genetic basis of DD in the proband. Segregation, in silico, and functional analyses were carried out to explore potential pathogenicity in the candidate mutation.

**Results:**

Exome sequencing and Sanger sequencing identified one novel missense mutation (p.G93R) in the *LAMP2* gene in the proband, and this mutation was also identified in three other family members. In silico analysis of *LAMP2* predicted that the mutation causes a conformational change and subsequent protein destabilization. Furthermore, functional examination showed that mutation carriers have a significant reduction in *LAMP2* expression, which supports that the mutation is pathogenic. Moreover, skewed X chromosome inactivation (XCI) was identified in one female mutation carrier, thus suggesting that skewed XCI may be the reason why this individual escaped the pathogenic influence of the mutation.

**Conclusion:**

These findings will aid in diagnosing DD patients carrying this *LAMP2* mutation that presents with a HCM phenotype. Furthermore, this study illustrates the importance of utilizing a molecular diagnostic approach in HCM patients and is the first study to report a *LAMP2* p.G93R mutation associated with mild DD and identify that XCI serves a protective role in DD etiology.

## 1 INTRODUCTION

Hypertrophic cardiomyopathy (HCM), clinically defined as a thickening of the myocardial wall in the absence of any other cause of left ventricular hypertrophy, is often inherited genetically. The etiology of familial HCM can be complex, with the majority of cases attributed to sarcomeric cardiomyopathies, mutations in sarcomere genes, and others associated with metabolic diseases, including Danon disease (DD), Pompe disease, or Fabry disease (Semsarian, Ingles, Maron, & Maron, 2015). DD is a rare monogenic X‐linked genetic disorder characterized by a clinical triad comprising cardiomyopathy, skeletal myopathy, and varying degrees of intellectual disability. The molecular basis of DD is attributed to an absence or malfunction of the lysosomal‐associated membrane protein 2 (*LAMP2*) due to pathogenic mutations in the *LAMP2* gene [OMIM:309060] (D'Souza et al., 2014). Thus, genetic testing can provide a more accurate clinical diagnosis, particularly for familial HCM cases, and aid in evaluating the risk of disease development and promote further HCM pathogenesis research.

In the present study, a proband presenting with a familial HCM phenotype was genetically evaluated using an exome sequencing approach to identify any pathogenic mutations. Two mutations, p.G93R in *LAMP2* and p.G790del in *DSC2* [OMIM:125645], were identified, and pathogenicity was explored using segregation, in silico, and functional analyses.

## 2 CASE STUDY

**Figure 1:**
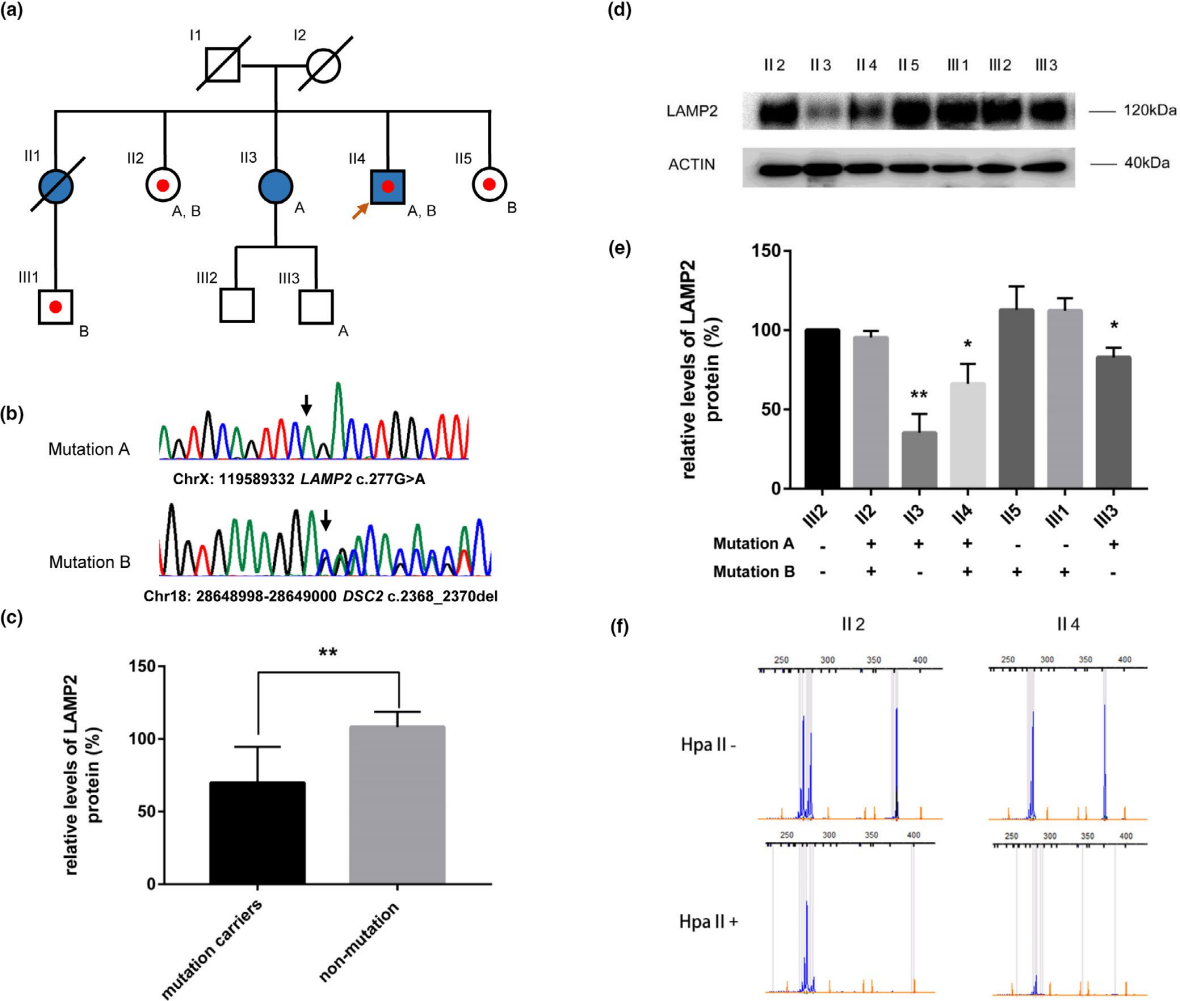
(a) Pedigree for the index patient. Squares = males; circles = females; filled shapes = HCM phenotype‐positive individuals; empty shapes = healthy individuals; shapes with a dot = ECG abnormality phenotype; an arrow indicates the proband; mutations are shown beside corresponding patients. (b) Direct sequencing electropherograms showing *LAMP2*_p.G93R (NM_013995.2) and *DSC2*_p.G790del (NM_024422.4) in the proband. Arrows indicate the mutant nucleotide positions. (c) Comparison of *LAMP2* expression levels between *LAMP2*_G93R mutation carriers and nonmutation subjects. (d) Western blot analysis of *LAMP2* expression in leukocyte extracts from the proband (II 4) and six family members (II 2, II 3, II 5, III 1, III 2, and III 3). (e) Relative *LAMP2* protein levels normalized to β‐actin for the family members. The *LAMP2* protein level in sample III 2 has been set to 100%. The data were quantified using ImageJ software and graphed using GraphPad Prism 7.0. *p < .01, **p < .001. (f) Analysis of X chromosome inactivation (XCI) patterns in leukocytes from individuals II 2 and II 4 (proband), with the proband's DNA used as a control. The *MIC2* gene is used to confirm complete digestion. Undigested (Hpa II−) and digested (Hpa II+) DNA samples are shown in the upper and lower plots, respectively. Comparisons of differences in the fluorescence intensities (heights of chromatographic peaks) corresponding to specific alleles (273 and 282 bp) after digestion indicates a skewed XCI pattern

In 2011, a 45‐year‐old man (Figure 1a; II 4) was referred to Southeast University Hospital due to repeated attacks of dizziness and chest pain. An echocardiogram revealed asymmetric septal hypertrophy and decreased left ventricular diastolic function. The left ventricular ejection fraction (LVEF) was 58% (Table 1). The interventricular septum (IVS) thickness was increased to 20 mm, and the left ventricular posterior wall (LVPW) thickness was 11 mm. An electrocardiograph (ECG) showed a sinus rhythm with ventricular pre‐excitation, a voltage criterion consistent with left ventricular hypertrophy, an ST segment depression, and a T wave inversion. Laboratory tests revealed normal serum creatine kinase (CK) activity levels. His eldest sister (II 1) died suddenly at the age of 63 (Figure 1a). Bisoprolol and diltiazem were used to improve symptoms.

**Table 1:** Clinical characteristics and echocardiogram and electrocardiograph findings for the family

Family member	II 2	II 3	II 4	II 5	III 1	III 2	III 3
Gender	F	F	M	F	M	M	M
Age	60	56	52	50	40	23	33
Genetic status	*LAMP2* (+) *DSC2* (+)	*LAMP2* (+) *DSC2* (−)	*LAMP2* (+) *DSC2* (+)	*LAMP2* (−) *DSC2* (+)	*LAMP2* (−) *DSC2* (+)	*LAMP2* (−) *DSC2* (−)	*LAMP2* (+) *DSC2* (−)
Cardiomyopathy	−	+	+	−	−	−	−
IVS	12	14	20	11	13	12	12
LVPW	12	14	14	10	13	11	12
E/A ratio	<1	<1	<1	<1	<1	>1	>1
LVEF	68%	55%	52%	64%	63%	72%	67%
ECG abnormality	+	−	+	+	+	−	−
Inverted T wave	+	−	+	+	+	−	−
Mental retardation	−	−	−	−	−	−	−
Muscle weakness	−	−	−	−	−	−	−
CK levels (U/L)	120	102	106	110	115	150	130

Abbreviations: −, no; +, yes; ECG, electrocardiograph; IVS, interventricular septum; LVEF, left ventricular ejection fraction; LVPW, left ventricular posterior wall.

In 2017, CK levels were elevated only once (260 U/L). In 2018 (7 years later), a follow‐up indicated that cardiomyopathy of the proband remained stable. The IVS thickness was unchanged, while the LVPW thickness was increased to 14 mm, and the LVEF was reduced to 52%. Furthermore, one sister (II 3) was diagnosed with HCM in 2013, with a maximum left ventricular wall thickness of 14 mm.

## 3 MATERIALS AND METHODS

### 3.1 Ethical compliance

Informed consent was obtained from the proband and family members prior to collecting blood samples or performing clinical evaluations. All protocols and procedures were approved by the Ethics Committee of Southeast University, Nanjing, Jiangsu Province, China.

### 3.2 Subjects and clinical evaluation

The relatives of the proband and the proband underwent a clinical examination, including evaluating CK levels and performing a 12‐lead ECG and two‐dimensional Doppler echocardiogram. Individuals identified as *LAMP2* mutation carriers were also invited for extensive neurologic and ophthalmologic examination.

### 3.3 Exome sequencing

The proband sample was examined using next‐generation sequencing (NGS), with enrichment performed using a cardio‐disease‐related Gene Panel (MyGenostics GenCap Enrichment Technologies). Exon and in exon‐intron boundaries for 178 genes commonly used for diagnosing cardiovascular diseases were selected and analyzed (Table S1). NGS was carried out on an Illumina HiSeq 2000 sequencer at a high‐quality sequencing depth (330.1). All targeted regions were mapped, which accounted for ∼99.8% of the 178 genes.

### 3.4 Data analysis

Low quality reads and adapters were filtered out using the Trim Galore program, and clean reads were aligned to the h19 human reference genome using the BWA program (Li & Durbin, 2010). Insertion–deletions and single nucleotide polymorphisms (SNPs) were identified using Sequence Alignment/Map tools (SAMtools) (Li et al., 2009) and detected using the Genome Analysis Toolkit (GATK) program. Annotations, including location (exonic, intronic, and intergenic region) and protein‐coding effect (synonymous, missense, nonsense, or frameshift), were obtained by using in‐house developed bioinformatics tools with RefSeq (hg19, from UCSC) and UCSC annotations as previously described (Xu et al., 2015). Candidate mutations for further analysis were determined based on frequency and function. For the frequency filter, a 0.01 cut‐off was used based on the allele frequency estimates from the dbSNP (v152; http://www.ncbi.nlm.nih.gov/projects/SNP/), 1,000 Genome (http://www.ncbi.nlm.nih.gov/Ftp/), and Exome Aggregation Consortium (ExAC; http://exac.broadinstitute.org/) databases. The functional filter removed synonymous and missense mutations that were predicted to be benign/tolerated using SIFT and Polyphen2 (Adzhubei et al., 2010; Kumar, Henikoff, & Ng, 2009).

Candidate variants were then further analyzed, with their potential impact and conservation scores examined using different computational programs, including MutationTaster (http://doro.charite.de/MutationTaster/index.html), MutationAssessor (http://mutationassessor.org/r3/), PROVEAN (http://provean.jcvi.org/seq_submit.php), FATHMM (http://fathmm.biocompute.org.uk/), FATHMM‐MKL (http://fathmm.biocompute.org.uk/fathmmMKL.html), likelihood ratio test (LRT; Chun & Fay, 2009), Combined Annotation Dependent Depletion (CADD; https://cadd.gs.washington.edu/score), genomic evolutionary rate profiling (GERP; Davydov et al., 2010), phastCons (Siepel et al., 2005), and SiPhy (Lindblad‐Toh et al., 2011).

### 3.5 Mutation validation

The identified mutations were confirmed using the Sanger sequencing method, with the proband and additional family members screened.

### 3.6 Western blot analysis

Western blotting was carried out following standard procedures (Mahmood & Yang, 2012). Briefly, total protein concentrations were determined using a Bradford assay. Samples containing equivalent amounts of protein were loaded onto 8% gels for SDS‐PAGE. The separated proteins were then transferred onto PVDF membranes. The membranes were blocked and then incubated with primary goat anti‐*LAMP2* polyclonal antibodies (R&D Systems) or mouse anti‐Actin monoclonal antibodies (Cell Signaling Technology). The membranes were then incubated with anti‐goat or anti‐mouse secondary antibodies (EMD Millipore) and visualized.

### 3.7 X chromosome inactivation pattern analysis

X chromosome inactivation (XCI) pattern analysis was used to isolate CpG dinucleotides with cytosine methylation that are located within the polymorphic CAG repeat of the human androgen receptor (AR) gene promoter region. The MIC promoter region (375 bp) was used to establish complete digestion, and the proband's DNA sample was included as a control. The DNA was digested with the methylation‐sensitive restriction enzyme HpaII (Takara, Japan). The PCR primers included: AR‐FP: 6‐FAM‐TCCAGAATCTGTTCCAGAGCGTGC, AR‐RP: GCTGTGAAGGTTGCTGTTCCTCAT, MIC2‐FP: 6‐FAM‐AGAGGTGCGTCC GATTTTTCCC, and MIC2‐RP: ACCGCCGCAGATGGACAATT. The amplicons were then sequenced using an ABI 3730 (Applied Biosystems Inc.) and analyzed using GeneMarker software (SoftGenetics). XCI status was determined by performing peak comparisons between the two AR alleles (different sizes) from digested and nondigested DNA aliquots for each sample (Jones, 2014).

### 3.8 Protein structure prediction and visualization

The I‐TASSER (iterative threading assembly refinement) program (Yang et al., 2015) utilizes template‐based modeling by making structural predictions based on fold homology. Herein, both the protein homology/analogy recognition V 2.0 (Phyre2; Kelley, Mezulis, Yates, Wass, & Sternberg, 2015) and I‐TASSER programs were utilized to investigate the effects of the candidate mutations. Additionally, three‐dimensional (3D) structures of the wild‐type and mutant proteins were generated using Pymol 2.1.0 (www.pymol.org). To predict how the missense mutation affects protein stability, STRUM (Quan, Lv, & Zhang, 2016) was utilized and based on the I‐TASSER model. The value of the free energy difference between the wild‐type and mutant (ΔΔG) was utilized to predict the protein stability due to particular amino acid changes.

### 3.9 Statistical analysis

All the data are expressed as a means ± SE. Comparisons of quantitative data were performed by using Student's *t* test, with *p* < .05 deemed statistically significant.

## 4 RESULTS

### 4.1 Identification of p.G93R in *LAMP2* and p.G790del in *DSC2* in the proband

To investigate a possible genetic cause associated with familial HCM, exome sequencing was utilized to examine 178 cardio‐disease‐associated genes in the proband. Following analysis, to include the use of sequencing chromatograms (Figure 1b), only two candidate mutations, *LAMP2*_p.G93R (NM_013995.2) and *DSC2*_p.G790del (NM_024422.4), were identified.

The identified *LAMP2* (rs727504953) missense mutation (p.G93R) is localized in a lumenal domain and has a minor allele frequency (MAF) of 5.7e‐5 according to the ExAC database. In a previous study, the p.G93R variant was reported to be a likely benign variant (ClinVar, Variation ID: 179562) based on the proposed variant assessment framework (Duzkale et al., 2013). Herein, conservation analysis showed that the locus is highly conserved across vertebrates, despite a lack of conservation across mammalians (Table 2). Furthermore, bioinformatics analysis examining whether the p.G93R variant is possibly deleterious had three analytical programs suggests a possibly damaging to damaging status, while three other programs indicated a neutral status (Table 2).

**Table 2:** Bioinformatics analysis of identified mutations

Variant	*LAMP2*_p.G93R	*DSC2*_p.G790del
SNP ID	rs727504953	rs377272752
Frequency in ExAC	5.7e−5	1.4e−3
SIFT_pred	**D**	**D**
Polyphen2_HDIV_pred	**P**	—
Polyphen2_HVAR_pred	B	—
MutationTaster_pred	**D**	**D**
MutationAssessor_pred	**M**	—
PROVEAN_pred	**D**	**D**
FATHMM_pred	T	—
FATHMM‐MKL_pred	**D**	—
LRT	N	—
CADD	11.92	
GERP	1.68	—
phastCons100way_vertebrate	**0.98**	—
phastCons20way_mammalian	0.12	
SiPhy_29way	6.025	—

Note

The table includes scores that assess pathogenicity and evolutionary conservation. The bold bioinformatic results indicate a potential deleterious impact for the variants. *LAMP2*_p.G93R (NM_013995.2), and *DSC2*_p.G790del (NM_024422.4). SIFT: D, deleterious; T, tolerated. Polyphen2: D, probably damaging; P, possibly damaging; B, benign. MutationTaster: D, disease causing; P, polymorphism. MutationAssessor: H, high; M, medium; L, low; N, neutral. PROVEAN: D, deleterious; N, neutral. FATHMM: D, deleterious; T, tolerated. FATHMM‐MKL: D, deleterious; T, tolerated. LRT: D, deleterious; N, neutral; U, unknown. CADD: P, pathogenic.

Abbreviations: CADD, combined annotation dependent depletion; ExAC, exome aggregation consortium; GERP, genomic evolutionary rate profiling; LRT, likelihood ratio test; SNP, single nucleotide polymorphism.

In the proband, one rare in‐frame deletion mutation, p.G790del, was identified in *DSC2* (rs377272752), with a MAF of about 0.14%. This deletion occurred at a highly conserved residue. However, the interpretations of pathogenicity were varied, from benign to likely pathogenic within the ClinVar database (Variation ID: 180319), to being predicted as damaging by SIFT, MutationTaster, and PROVEAN databases.

### 4.2 Genotype–phenotype correlation

The identification of the p.G93R mutation in *LAMP2* can be associated with a DD diagnosis. Since DD may also involve skeletal myopathy, cognitive disorders, and visual disturbances, the proband was referred to a neurologist and an ophthalmologist for a formal assessment. His CK levels were normal (106 U/L), and both the neurological and ophthalmologic examinations were normal, with no neuromuscular abnormalities detected following electromyography.

Further genetic screening was performed for various relatives and revealed that the *LAMP2* p.G93R mutation can be found in hemizygotic males (II 4 and III 3) and heterozygotic females (II 2 and II 3), while the *DSC2* p.G790del mutation was only found in individuals II 2, II 4, II 5, and III 1 (Figure 1a). No toxic exposure was reported among the family members. Clinical evaluations were also performed for the evaluated family members, and all members had normal serum CK levels. Echocardiograms showed that family members II 3 and II 4 had left ventricular diastolic dysfunction with LVEFs at 55% and 52%, respectively. Twelve‐lead ECGs showed that varying degrees of T inversion were present in individuals II 2, II 4, II 5, and III 1, all of whom are *DSC2*_ p.G790del mutation carriers (Table 1).

### 4.3 Structural analysis of *LAMP2* p.G93R and *DSC2* p.G790del mutations

**Figure 2:**
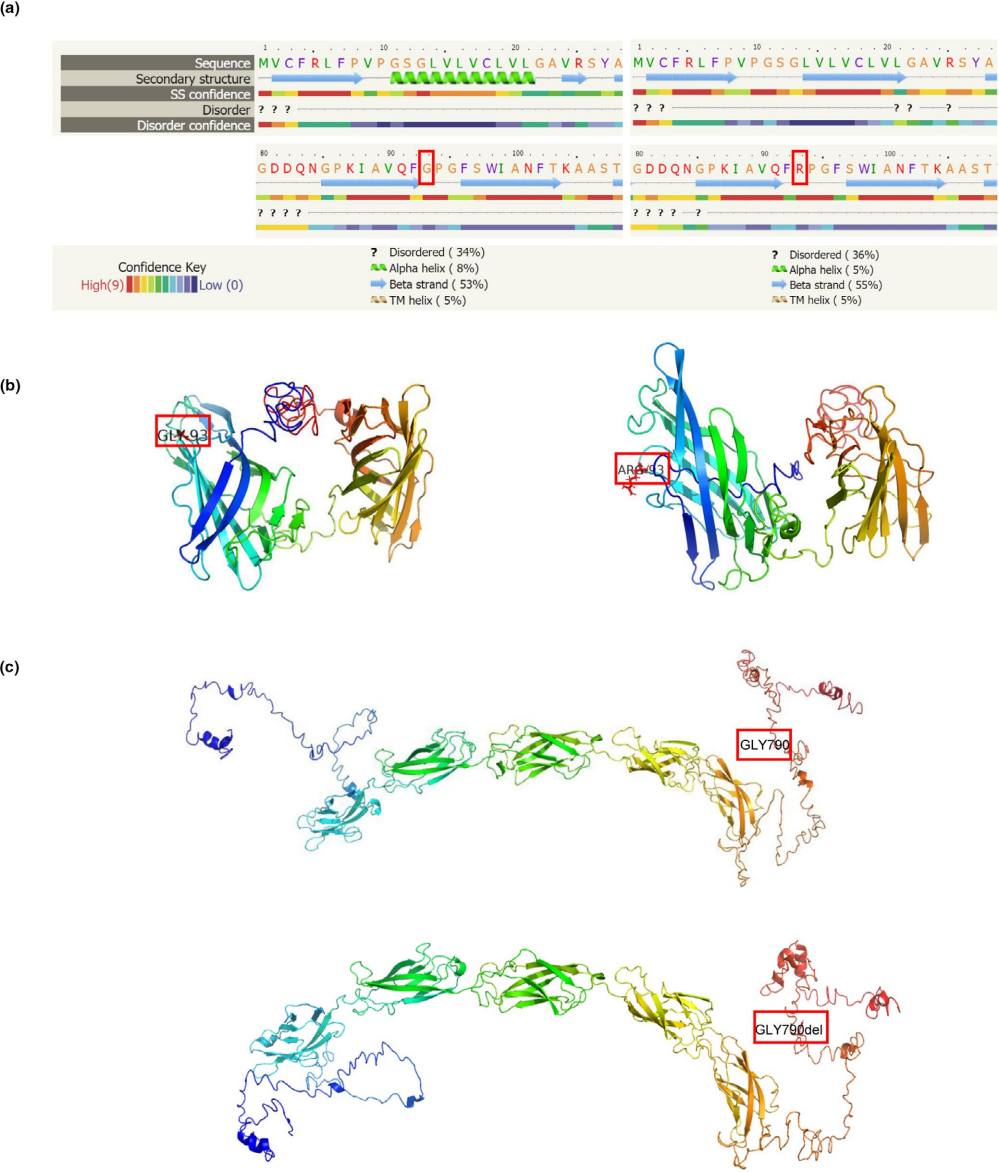
(a) Predicted secondary structure based on template/homology modeling using Phyre2. The first line indicates the amino acid sequence, and the second line is the secondary structure prediction for the normal *LAMP2* protein (left) and the mutant protein (Arg93) (right), with the high confidence value score indicated with light red on line three. The secondary structure around the N‐terminal is affected in the mutant protein. (b) *LAMP2* 3D structure for the wild‐type (left) and Gly93Arg mutant (right) as generated with PyMOL. The mutation site is highlighted in red. (c) DSC2 3D structure for the wild‐type and Gly790del mutant

To elucidate the mutational consequences, structural analysis for both the *LAMP2* p.G93R and *DSC2* p.G790del mutations was performed. Full‐length *LAMP2* (UniProtKB/Swiss‐Prot P13473) and DSC2 (UniProtKB/Swiss‐Prot Q02487) amino acid sequences were imported into the I‐TASSER program, and the best output model was selected based on the internal ranking. The threading templates most frequently used by I‐TASSER to construct the *LAMP2* model were obtained from the Protein Data Bank (PDB; accession numbers: 5gv3A, 4akmA, 5gv0A, and 5gv3). A high secondary structure prediction confidence score was obtained for the Gly93 position, with this mutation predicted to cause an N‐terminal change from an alpha‐helical structure to a beta strand, which is consistent with the Phyre2 results (Figure 2a). *LAMP2* wild‐type and p.G93R mutant 3D models were constructed using Pymol (Figure 2b), with differences noted that appear to be reflecting different physical and chemical properties. Furthermore, when evaluating the protein stability based on the obtained model using STRUM (Quan et al., 2016), ΔΔG = −1.13 was obtained, thus indicating that the mutation causes destabilization.

The top threading template for predicting both the DSC2 p.G790del and normal models was based on PDB accession number 5iryA (human Desmocollin‐1 ectodomain), with an identity = 0.55, normalized Z‐score = 3.44, and coverage = 0.60. Moreover, based on the constructed model, the p.G790del mutation is predicted to affect the C‐terminal secondary structure, subsequently causing a drastic conformational change in the cytoplasmic tail of DSC2 (Figure 2c).

### 4.4 *LAMP2* p.G93R mutation significantly reduces *LAMP2* expression

To assess the functional effect of the p.G93R mutation, *LAMP2* expression in peripheral white blood cells was examined in family members (II 2, II 3, II 4, II 5, III 1, III 2, and III 3) via Western blotting. Three independent experiments were quantified and graphed (Figure 1c–e), and relative *LAMP2* protein levels were compared between mutation carriers and nonmutation subjects. The results showed a significant difference in *LAMP2* expression between *LAMP2* mutation carriers and nonmutation subjects (Figure 1c), while no significant difference in *LAMP2* expression was noted between *DSC2* mutation carriers and nonmutation subjects (data not shown). Moreover, of the four family members carrying the *LAMP2* p.G93R mutation, *LAMP2* protein expression levels were only significantly decreased in three of the *LAMP2* mutation carriers (II 3, II 4, and III 3) when compared with the nonmutation carrier (III 2), with no significant difference noted in the remaining *LAMP2* mutation carrier (II 2; Figure 1d,e).

### 4.5 Skewed X chromosome inactivation in one female p.G93R mutation carrier

To explore the possibility that the absence of clinical signs and a *LAMP2* protein reduction in a female mutation carrier (II 2) is attributable to a skewed XCI, inactivation analysis was performed. Sufficient HpaII digestion of the active allele was confirmed by the absence of a corresponding peak area in a male sample. The results for the II 2 individual showed a skewing at levels of 82%:18%, thus indicating a skewed XCI pattern (Figure 1d). Furthermore, females II 3 and II 5 were found to be homozygous for the polymorphic CAG repeat, thus could not provide any additional insight.

## 5 DISCUSSION

Recently developed exome sequencing technologies and bioinformatics tools offer cost effective and highly accurate results and have been widely used to identify genetically inherited diseases. Herein, the genetic underpinnings associated with a familial HCM case were examined by applying exome sequencing. After examining the proband, *LAMP2*_ p.G93R and *DSC2*_p.G790del mutations were identified as presenting with an asymmetric HCM phenotype. In this study, the patient's disease presentation, as well as the disease course, is the mildest DD case yet identified. Furthermore, of the four family members carrying the *LAMP2* p.G93R mutation, only one male member and one female member showed a mild HCM phenotype regarding left ventricular diastolic dysfunction. Both of these individuals did not suffer from all of the clinical triad commonly associated with this disease as described above. Additionally, in silico analysis predicted that the *LAMP2* p.G93R mutation causes a structural change that leads to protein instability. Furthermore, functional evaluation showed that in *LAMP2* p.G93R mutation carriers, *LAMP2* expression is reduced when compared with nonmutation carriers. These findings suggest that this genetic mutation is in fact pathogenic.

A skewed XCI in the peripheral blood of patients with X‐linked diseases has been previously documented (Vacca, Della, Scalabri, & D'Esposito, 2016). Herein, DD was found to be caused by heterozygous mutations in an X‐linked gene, thus suggesting that XCI might have a role in DD and contribute to the clinical phenotype variability. In this study, one female *LAMP2* p.G93R mutation carrier (II 2) was found to have a skewed XCI. She showed a normal left ventricle and nearly normal *LAMP2* expression, whereas the other female mutation carrier (II 3) showed a hypertrophic left ventricle, left ventricular diastolic dysfunction and decreased *LAMP2* expression. It is likely that the skewed XCI ratio found in female II 2 helped her escape the negative influence of the mutation and offered protection from DD. In another study, a very different outcome was reported with an early onset DD female carrying a *LAMP2* c.453delT mutation given an unfavorable prognosis (Bottillo et al., 2016). In the II 2 female in this study, an uneven and patchy distribution of *LAMP2* was noted in her cardiomyocytes, which correlated with the percentage of XCI in her blood leukocytes and cardiac muscle. All together, these findings highlight the importance of XCI in DD etiology.

Notably, over 70 mutations have been reported to be associated with DD (D'Souza et al., 2014), with most being loss of function mutations, including frameshift, nonsense, and splicing mutations. To date, only three of the reported mutations have been missense mutations (p.W321R, p.G384R, and p.C18R) (Kyaw, Shaik, Lin, & Shinnar, 2018; Musumeci et al., 2005; van der Kooi et al., 2008). Furthermore, all of these missense mutations occur in *LAMP2* and are associated with atypical and late onset DD. Mental retardation, while common in DD, is not observed in these patients. Patients carrying the p.W321R mutation present with HCM, persistent hyperCKemia, and exercise intolerance (Musumeci et al., 2005). However, in this study, the proband and family members carrying the p.G384R mutation were characterized by left ventricular hypertrophy, progressive muscle weakness (van der Kooi et al., 2008), and cone‐rod dystrophy (Thiadens et al., 2012). Additionally, another study examining one patient carrying the p.C18R mutation reported the individual to have severe HCM and severe dilated cardiomyopathy with an absence of neurological and skeletal muscle pathology (Kyaw et al., 2018). Interestingly, a previous study also identified the *LAMP2* p.G93R mutation in one proband with familial HCM but reported it to likely be a benign variant due to a lack of conservation (Duzkale et al., 2013) (Laboratory for Molecular Medicine, Partners HealthCare Personalized Medicine). These discrepancies will require further examination in future studies to resolve these differences.

Usually, DD is a rapidly progressing and fatal disease. Only very few DD cases present with only a cardiac phenotype in the absence of other symptoms (Dougu et al., 2009; Hedberg et al., 2015; Kim et al., 2014). Sugie et al. described a female DD patient with decreased *LAMP2* expression and proposed that a 50% reduction in *LAMP2* expression may prevent the development of myopathy, but not cardiomyopathy (Sugie et al., 2018). Therefore, we also propose that phenotypes restricted to heart involvement may be attributed to a mutated *LAMP2* protein having some residual function. However, less than 50% of *LAMP2* expression was detected in the II 3 individual; thus the absence of myopathy may be attributed to other factors as well.

*DSC2*, encoding desmocollin 2, has been reported to be associated with arrhythmogenic right ventricular cardiomyopathy. In the present family, an in‐frame deletion mutation, *DSC2*_p.G790del, was also identified. Notably, this p.G790del mutation has been previously reported in two patients with epilepsy‐related sudden unexpected death (Hata, Yoshida, Kinoshita, & Nishida, 2017). Fibrofatty replacement of the right ventricle was found in both patients, suggesting a possible causative role associated with the mutation. In this study, despite conflicting interpretations in the ClinVar database, the in silico analysis predicted that the deletion variant disrupts the cytoplasmic tail. Moreover, a T wave inversion was present in the *DSC2* mutation carriers, which indicates that the p.G790del variant can affect phenotypic expression. Thus, it would seem that this variant probably acts as a possible candidate genetic modifier. However, more genetic evidence and functional experiments are needed for confirmation.

Based on previous reports and the findings presented herein, *LAMP2* haploinsufficiency, along with XCI, is most likely the important determinant of phenotype in female patients. Other mechanisms, such as genetic modifiers and lifestyle, may also be associated with variable phenotypic expression. Still, the establishment of the pathogenesis underlying DD requires further investigation.

## 6 CONCLUSION

The findings presented herein suggest that the *LAMP2* p.G93R mutation causes mild DD that presents with late onset familial HCM. This study broadens the spectrum of DD, with this study reporting the first case where XCI serves a protective role in one female mutation carrier. Considering the mild disease presentation, DD may be overlooked in such HCM patients. Genetic analysis, along with a correct interpretation of the genetic findings, is necessary to provide an accurate diagnosis and is of the utmost importance for better patient management. The approach utilized herein should be a mainstay diagnostic tool, particularly when attempting to obtain a differential diagnosis of disorders with a HCM phenotype.

## CONFLICT OF INTEREST

The authors have no conflict of interest to declare.
