# Supplementary material for: A novel LAMP2 p.G93R mutation associated with mild Danon disease presenting with familial hypertrophic cardiomyopathy
Source: Mol Genet Genomic Med. 2019 Aug 28;7(10):e00941. doi: 10.1002/mgg3.941 (PMC6785429; doi:10.1002/mgg3.941)
Supplement: Supplementary file 1 [file MGG3-7-e00941-s001.docx]

Table S1. List of 178 cardio-disease-associated genes utilized for exome sequencing

| Gene | Locus | Description | OMIM |
| --- | --- | --- | --- |
| *AARS2* | 6p21.1 | alanyl-tRNA synthetase 2, mitochondrial | 612035 |
| *ABCC1* | 16p13.1 | ATP binding cassette subfamily C member 1 | 158343 |
| *ABCC9* | 12p12.1 | ATP binding cassette subfamily C member 9 | 601439 |
| *ACADS* | 12q24.31 | acyl-CoA dehydrogenase, C-2 to C-3 short chain | 606885 |
| *ACTC1* | 15q14 | actin, alpha, cardiac muscle 1 | 102540 |
| *ACTN1* | 14q24; 14q22-q24 | actinin, alpha 1 | 102575 |
| *ACTN2* | 1q42-q43 | actinin, alpha 2 | 102573 |
| *ACVRL1* | 12q13.13 | activin A receptor type IL | 601284 |
| *ADRA2C* | 4p16 | adrenoceptor alpha 2C | 104250 |
| *ADRB1* | 10q25.3 | adrenoceptor beta 1 | 109630 |
| *AGTR2* | Xq22-q23 | angiotensin II receptor, type 2 | 300034 |
| *AKAP9* | 7q21-q22 | A-kinase anchoring protein 9 | 604001 |
| *ANGPT1* | 8q23.1 | angiopoietin 1 | 601667 |
| *ANK2* | 4q25-q27 | ankyrin 2, neuronal | 106410 |
| *ANKRD1* | 10q23.31 | ankyrin repeat domain 1 (cardiac muscle) | 609599 |
| *BAG3* | 10q25.2-q26.2 | BCL2 associated athanogene 3 | 603883 |
| *BMP2* | 20p12 | bone morphogenetic protein 2 | 112261 |
| *BMP4* | 14q22-q23 | bone morphogenetic protein 4 | 112262 |
| *BMPR1A* | 10q22.3 | bone morphogenetic protein receptor type IA | 601299 |
| *BMPR1B* | 4q22-q24 | bone morphogenetic protein receptor type IB | 603248 |
| *BMPR2* | 2q33-q34 | bone morphogenetic protein receptor type II | 600799 |
| *BRAF* | 7q34 | B-Raf proto-oncogene, serine/threonine kinase | 164757 |
| *CACNA1C* | 12p13.3 | calcium channel, voltage-dependent, L type, alpha 1C subunit | 114205 |
| *CACNB2* | 10p12 | calcium channel, voltage-dependent, beta 2 subunit | 600003 |
| *CASQ2* | 1p13.1 | calsequestrin 2 | 114251 |
| *CAV3* | 3p25 | caveolin 3 | 601253 |
| *CBL* | 11q23.3 | Cbl proto-oncogene, E3 ubiquitin protein ligase | 165360 |
| *CFTR* | 7q31.2 | cystic fibrosis transmembrane conductance regulator | 602421 |
| *COL11A2* | 6p21.3 | collagen, type XI, alpha 2 | 120290 |
| *COL6A1* | 21q22.3 | collagen, type VI, alpha 1 | 120220 |
| *CRYAB* | 11q23.1 | crystallin alpha B | 123590 |
| *CSRP3* | 11p15.1 | cysteine and glycine rich protein 3 | 600824 |
| *DES* | 2q35 | desmin | 125660 |
| *DMD* | Xp21.2 | dystrophin | 300377 |
| *DSC2* | 18q12.1 | desmocollin 2 | 125645 |
| *DSG2* | 18q12.1 | desmoglein 2 | 125671 |
| *DSG3* | 18q12.1 | desmoglein 3 | 169615 |
| *DSP* | 6p24 | desmoplakin | 125647 |
| *DTNA* | 18q12 | dystrobrevin alpha | 601239 |
| *EMD* | Xq28 | emerin | 300384 |
| *ENG* | 9q34.11 | endoglin | 131195 |
| *ENPP1* | 6q22-q23 | ectonucleotide pyrophosphatase/phosphodiesterase 1 | 173335 |
| *EYA4* | 6q23 | EYA transcriptional coactivator and phosphatase 4 | 603550 |
| *F12* | 5q35.3 | coagulation factor XII (Hageman factor) | 610619 |
| *F13A1* | 6p25.3-p24.3 | coagulation factor XIII, A1 polypeptide | 134570 |
| *F2* | 11p11 | coagulation factor II (thrombin) | 176930 |
| *F5* | 1q23 | coagulation factor V (proaccelerin, labile factor) | 612309 |
| *F7* | 13q34 | coagulation factor VII (serum prothrombin conversion accelerator) | 613878 |
| *F9* | Xq27.1-q27.2 | coagulation factor IX | 300746 |
| *FADS1* | 11q12.2-q13.1 | fatty acid desaturase 1 | 606148 |
| *FAM13A* | 4q22.1 | family with sequence similarity 13 member A | 613299 |
| *FGA* | 4q28 | fibrinogen alpha chain | 134820 |
| *FGB* | 4q28 | fibrinogen beta chain | 134830 |
| *FGF2* | 4q26 | fibroblast growth factor 2 (basic) | 134920 |
| *FGFR1* | 8p11.23-p11.22 | fibroblast growth factor receptor 1 | 136350 |
| *FGFR2* | 10q26 | fibroblast growth factor receptor 2 | 176943 |
| *FGG* | 4q28 | fibrinogen gamma chain | 134850 |
| *FHL2* | 2q12.2 | four and a half LIM domains 2 | 602633 |
| *FHOD3* | 18q12 | formin homology 2 domain containing 3 | 609691 |
| *FKTN* | 9q31.2 | fukutin | 607440 |
| *GATAD1* | 7q21-q22 | GATA zinc finger domain containing 1 | 614518 |
| *GDF2* | 10q11.22 | growth differentiation factor 2 | 605120 |
| *GLA* | Xq22 | galactosidase alpha | 300644 |
| *GPD1L* | 3p22.3 | glycerol-3-phosphate dehydrogenase 1-like | 611778 |
| *GSTM1* | 1p13.3 | glutathione S-transferase mu 1 | 138350 |
| *HABP2* | 10q25.3 | hyaluronan binding protein 2 | 603924 |
| *HCN4* | 15q24.1 | hyperpolarization activated cyclic nucleotide gated potassium channel 4 | 605206 |
| *HLA-B* | 6p21.3 | major histocompatibility complex, class I, B | 142830 |
| *HLA-DPB1* | 6p21.3 | major histocompatibility complex, class II, DP beta 1 | 142858 |
| *HRG* | 3q27 | histidine-rich glycoprotein [Homo sapiens | 142640 |
| *HTR2B* | 2q36.3-q37.1 | 5-hydroxytryptamine (serotonin) receptor 2B, G protein-coupled | 601122 |
| *IFT172* | 2p23.3 | intraflagellar transport 172 | 607386 |
| *ILK* | 11p15.4 | integrin linked kinase | 602366 |
| *IREB2* | 15q25.1 | iron responsive element binding protein 2 | 147582 |
| *JPH2* | 20q13.12 | junctophilin 2 | 605267 |
| *JUP* | 17q21 | junction plakoglobin | 173325 |
| *KCNA5* | 12p13 | potassium channel, voltage gated shaker related subfamily A, member 5 | 176267 |
| *KCNE1* | 21q22.12 | potassium channel, voltage gated subfamily E regulatory beta subunit 1 | 176261 |
| *KCNE2* | 21q22.12 | potassium channel, voltage gated subfamily E regulatory beta subunit 2 | 603796 |
| *KCNE3* | 11q13.4 | potassium channel, voltage gated subfamily E regulatory beta subunit 3 | 604433 |
| *KCNH2* | 7q36.1 | potassium channel, voltage gated eag related subfamily H, member 2 | 152427 |
| *KCNJ2* | 17q24.3 | potassium channel, inwardly rectifying subfamily J, member 2 | 600681 |
| *KCNJ5* | 11q24 | potassium channel, inwardly rectifying subfamily J, member 5 | 600734 |
| *KCNQ1* | 11p15.5 | potassium channel, voltage gated KQT-like subfamily Q, member 1 | 607542 |
| *KRAS* | 12p12.1 | Kirsten rat sarcoma viral oncogene homolog | 190070 |
| *LAMP2* | Xq24 | lysosomal-associated membrane protein 2 | 309060 |
| *LDB3* | 10q22.3-q23.2 | LIM domain binding 3 | 605906 |
| *LMNA* | 1q22 | lamin A/C | 150330 |
| *MMP12* | 11q22.3 | matrix metallopeptidase 12 | 601046 |
| *MTHFR* | 1p36.3 | methylenetetrahydrofolate reductase (NAD(P)H) | 607093 |
| *MUC2* | 11p15.5 | mucin 2, oligomeric mucus/gel-forming | 158370 |
| *MUC5B* | 11p15.5 | mucin 5B, oligomeric mucus/gel-forming | 600770 |
| *MYBPC3* | 11p11.2 | myosin binding protein C, cardiac | 600958 |
| *MYH6* | 14q12 | myosin, heavy chain 6, cardiac muscle, alpha | 160710 |
| *MYH7* | 14q12 | myosin, heavy chain 7, cardiac muscle, beta | 160760 |
| *MYL2* | 12q24.11 | myosin light chain 2 | 160781 |
| *MYL3* | 3p21.3-p21.2 | myosin, light chain 3, alkali; ventricular, skeletal, slow | 160790 |
| *MYLK2* | 20q13.31 | myosin light chain kinase 2 | 606566 |
| *MYOZ2* | 4q26-q27 | myozenin 2 | 605602 |
| *MYPN* | 10q21.3 | myopalladin | 608517 |
| *NEXN* | 1p31.1 | nexilin (F actin binding protein) | 613121 |
| *NFKBIL1* | 6p21.3 | nuclear factor of kappa light polypeptide gene enhancer in B-cells inhibitor-like 1 | 601022 |
| *NOS3* | 7q36 | nitric oxide synthase 3 | 163729 |
| *NRAS* | 1p13.2 | neuroblastoma RAS viral (v-ras) oncogene homolog | 164790 |
| *OBSCN* | 1q42.13 | obscurin, cytoskeletal calmodulin and titin-interacting RhoGEF | 608616 |
| *PDLIM3* | 4q35 | PDZ and LIM domain 3 | 605889 |
| *PIGA* | Xp22.1 | phosphatidylinositol glycan anchor biosynthesis class A | 311770 |
| *PKP2* | 12p11 | plakophilin 2 | 602861 |
| *PLAT* | 8p12 | plasminogen activator, tissue | 173370 |
| *PLG* | 6q26 | plasminogen [Homo sapiens | 173350 |
| *PLN* | 6q22.1 | phospholamban | 172405 |
| *PRKAG2* | 7q36.1 | protein kinase, AMP-activated, gamma 2 non-catalytic subunit | 602743 |
| *PROC* | 2q13-q14 | protein C, inactivator of coagulation factors Va and VIIIa | 612283 |
| *PROCR* | 20q11.2 | protein C receptor | 600646 |
| *PROS1* | 3q11.2 | protein S (alpha) | 176880 |
| *PSEN1* | 14q24.3 | presenilin 1 | 104311 |
| *PSEN2* | 1q42.13 | presenilin 2 | 600759 |
| *PTPN11* | 12q24 | protein tyrosine phosphatase, non-receptor type 11 | 176876 |
| *RAF1* | 3p25 | Raf-1 proto-oncogene, serine/threonine kinase | 164760 |
| *RBM20* | 10q25.2 | RNA binding motif protein 20 [Homo sapiens | 613171 |
| *RYR2* | 1q43 | ryanodine receptor 2 (cardiac) | 180902 |
| *SCD* | 10q24.31 | stearoyl-CoA desaturase (delta-9-desaturase) | 604031 |
| *SCN1B* | 19q13.1 | sodium channel, voltage gated, type I beta subunit | 600235 |
| *SCN3B* | 11q23.3 | sodium channel, voltage gated, type III beta subunit | 608214 |
| *SCN4B* | 11q23.3 | sodium channel, voltage gated, type IV beta subunit | 608256 |
| *SCN5A* | 3p21 | sodium channel, voltage gated, type V alpha subunit | 600163 |
| *SCO2* | 22q13.33 | SCO2 cytochrome c oxidase assembly protein | 604272 |
| *SDHC* | 1q23.3 | succinate dehydrogenase complex, subunit C, integral membrane protein, 15kDa | 602413 |
| *SERPINA1* | 14q32.1 | serpin peptidase inhibitor, clade A (alpha-1 antiproteinase, antitrypsin), member 1 | 107400 |
| *SERPINA10* | 14q32.13 | serpin peptidase inhibitor, clade A (alpha-1 antiproteinase, antitrypsin), member 10 | 605271 |
| *SERPINA3* | 14q32.1 | serpin peptidase inhibitor, clade A (alpha-1 antiproteinase, antitrypsin), member 3 | 107280 |
| *SERPINC1* | 1q25.1 | serpin peptidase inhibitor, clade C (antithrombin), member 1 | 107300 |
| *SERPIND1* | 22q11.21 | serpin peptidase inhibitor, clade D (heparin cofactor), member 1 | 142360 |
| *SFTPA1* | 10q22.3 | surfactant protein A1 | 178630 |
| *SFTPA2* | 10q22.3 | surfactant protein A2 | 178642 |
| *SGCD* | 5q33-q34 | sarcoglycan delta | 601411 |
| *SHOC2* | 10q25 | SHOC2 leucine-rich repeat scaffold protein | 602775 |
| *SLC16A9* | 10q21.2 | solute carrier family 16 member 9 | 614242 |
| *SLC25A4* | 4q35 | solute carrier family 25 (mitochondrial carrier; adenine nucleotide translocator), member 4 | 103220 |
| *SLC6A4* | 17q11.2 | solute carrier family 6 (neurotransmitter transporter), member 4 | 182138 |
| *SMAD1* | 4q31 | SMAD family member 1 | 601595 |
| *SMAD2* | 18q21.1 | SMAD family member 2 | 601366 |
| *SMAD3* | 15q22.33 | SMAD family member 3 | 603109 |
| *SMAD4* | 18q21.1 | SMAD family member 4 | 600993 |
| *SMAD5* | 5q31 | SMAD family member 5 | 603110 |
| *SMAD6* | 15q22.31 | SMAD family member 6 | 602931 |
| *SMAD7* | 18q21.1 | SMAD family member 7 | 602932 |
| *SMAD8* | 13q12-q14 | SMAD family member 9 | 603295 |
| *SNTA1* | 20q11.2 | syntrophin, alpha 1 | 601017 |
| *SOD3* | 4p15.2 | superoxide dismutase 3, extracellula | 185490 |
| *SOS1* | 2p21 | SOS Ras/Rac guanine nucleotide exchange factor 1 | 182530 |
| *TAZ* | Xq28 | tafazzin | 300394 |
| *TCAP* | 17q12 | titin-cap | 604488 |
| *TERC* | 3q26 | telomerase RNA component | 602322 |
| *TERT* | 5p15.33 | telomerase reverse transcriptase | 187270 |
| *TFPI* | 2q32 | tissue factor pathway inhibitor | 152310 |
| *TGFB1* | 19q13.1 | transforming growth factor beta 1 | 190180 |
| *TGFB3* | 14q24 | transforming growth factor beta 3 | 190230 |
| *TGFBR2* | 3p22 | transforming growth factor beta receptor II | 190182 |
| *THBD* | 20p11.2 | thrombomodulin | 188040 |
| *THBS1* | 15q15 | thrombospondin 1 | 188060 |
| *TMEM43* | 3p25.1 | transmembrane protein 43 | 612048 |
| *TMEM70* | 8q21.11 | transmembrane protein 70 | 612418 |
| *TMPO* | 12q22 | thymopoietin | 188380 |
| *TNF* | 6p21.3 | tumor necrosis factor | 191160 |
| *TNNC1* | 3p21.1 | troponin C type 1 (slow) | 191040 |
| *TNNC2* | 20q12-q13.11 | troponin C type 2 (fast) | 191039 |
| *TNNI1* | 1q31.3 | troponin I type 1 (skeletal, slow) | 191042 |
| *TNNI3* | 19q13.4 | troponin I type 3 (cardiac) | 191044 |
| *TNNT1* | 19q13.4 | troponin T type 1 (skeletal, slow) | 191041 |
| *TNNT2* | 1q32 | troponin T type 2 (cardiac) | 191045 |
| *TNNT3* | 11p15.5 | troponin T type 3 (skeletal, fast) | 600692 |
| *TPM1* | 15q22.1 | tropomyosin 1 (alpha) | 191010 |
| *TRPC6* | 11q22.1 | transient receptor potential cation channel, subfamily C, member 6 | 603652 |
| *TRPV4* | 12q24.1 | transient receptor potential cation channel, subfamily V, member 4 | 605427 |
| *TTN* | 2q31 | titin | 188840 |
| *TTR* | 18q12.1 | transthyretin | 176300 |
| *VCL* | 10q22.2 | vinculin | 193065 |
